# Association of Exposure to Ambient Air Pollution With Thyroid Function During Pregnancy

**DOI:** 10.1001/jamanetworkopen.2019.12902

**Published:** 2019-10-16

**Authors:** Akhgar Ghassabian, Livia Pierotti, Mikel Basterrechea, Leda Chatzi, Marisa Estarlich, Ana Fernández-Somoano, Abby F. Fleisch, Diane R. Gold, Jordi Julvez, Polyxeni Karakosta, Aitana Lertxundi, Maria-Jose Lopez-Espinosa, Tessa A. Mulder, Tim I. M. Korevaar, Emily Oken, Robin P. Peeters, Sheryl Rifas-Shiman, Euripides Stephanou, Adonina Tardón, Henning Tiemeier, Martine Vrijheid, Tanja G. M. Vrijkotte, Jordi Sunyer, Mònica Guxens

**Affiliations:** 1 Departments of Pediatrics, Environmental Medicine, and Population Health, School of Medicine, New York University, New York; 2 ISGlobal, Barcelona, Spain; 3 Spanish Consortium for Research on Epidemiology and Public Health, Madrid, Spain; 4 Universitat Pompeu Fabra, Barcelona, Spain; 5 Biodonostia Health Research Institute, San Sebastian, Spain; 6 Public Health Division of Gipuzkoa, Basque Government, San Sebastian, Spain; 7 Department of Social Medicine, University of Crete, Heraklion, Greece; 8 Department of Preventive Medicine, Keck School of Medicine, University of Southern California, Los Angeles; 9 Epidemiology and Environmental Health Joint Research Unit, The Foundation for the Promotion of Health and Biomedical Research of Valencia Region, Universitat Jaume I-Universitat de València, Valencia, Spain; 10 Instituto Universaitario de Oncología del Principado de Asturias, Departament of Medicine, University of Oviedo, Oviedo, Spain; 11 Department of Pediatric Endocrinology and Diabetes, Maine Medical Center, Portland; 12 Center for Outcomes Research and Evaluation, Maine Medical Center Research Institute, Portland; 13 Channing Division of Network Medicine, Brigham and Women’s Hospital, Harvard Medical School, Boston, Massachusetts; 14 Department of Environmental Health, Harvard T.H. Chan School of Public Health, Boston, Massachusetts; 15 Hospital del Mar Research Institute, Barcelona, Spain; 16 Clinical Microbiology Laboratory, Attikon University Hospital, Medical School, National and Kapodistrian University of Athens, Athens, Greece; 17 Department of Public Health and Preventive Medicine, University of Basque Country, Bilbao, Spain; 18 Academic Center for Thyroid Diseases, Department of Internal Medicine, Erasmus Medical Center, Rotterdam, the Netherlands; 19 Department of Child and Adolescent Psychiatry, Erasmus Medical Center–Sophia Children’s Hospital, Rotterdam, the Netherlands; 20 Division of Chronic Disease Research Across the Lifecourse, Department of Population Medicine, Harvard Medical School and Harvard Pilgrim Health Care Institution, Boston, Massachusetts; 21 Department of Nutrition, Harvard T.H. Chan School of Public Health, Boston, Massachusetts; 22 Department of Chemistry, University of Crete, Heraklion, Greece; 23 Department of Epidemiology, Harvard T.H. Chan School of Public Health, Boston, Massachusetts; 24 Department of Public Health, Amsterdam Public Health Research Institute, Amsterdam University Medical Center, University of Amsterdam, Amsterdam, the Netherlands

## Abstract

**Importance:**

Air pollutants interact with estrogen nuclear receptors, but their effect on thyroid signaling is less clear. Thyroid function is of particular importance for pregnant women because of the thyroid’s role in fetal brain development.

**Objective:**

To determine the short-term association of exposure to air pollution in the first trimester with thyroid function throughout pregnancy.

**Design, Setting, and Participants:**

In this cohort study, 9931 pregnant women from 4 European cohorts (the Amsterdam Born Children and Their Development Study, the Generation R Study, Infancia y Medio Ambiente, and Rhea) and 1 US cohort (Project Viva) with data on air pollution exposure and thyroid function during pregnancy were included. The recruitment period for the Amsterdam Born Children and Their Development Study was January 2003 to March 2004; for Generation R, April 2002 to January 2006; for Infancia y Medio Ambiente, November 2003 to January 2008; for Rhea, February 2007 to February 2008; and for Project Viva, April 1999 to November 2002. Statistical analyses were conducted from January 2018 to April 2019.

**Main Outcomes and Measures:**

Residential air pollution concentrations (ie, nitrogen oxide and particulate matter [PM]) during the first trimester of pregnancy were estimated using land-use regression and satellite-derived aerosol optical depth models. Free thyroxine, thyrotropin, and thyroid peroxidase antibody levels were measured across gestation. Hypothyroxinemia was defined as free thyroxine below the fifth percentile of the cohort distribution with normal thyrotropin levels, following the American Thyroid Association guidelines.

**Results:**

Among 9931 participants, the mean (SD) age was 31.2 (4.8) years, 4853 (48.9%) had more than secondary educational levels, 5616 (56.6%) were nulliparous, 404 (4.2%) had hypothyroxinemia, and 506 (6.7%) tested positive for thyroid peroxidase antibodies. Concentrations of nitrogen dioxide and PM with an aerodynamic diameter of 2.5 μm or less (PM_2.5_) were lower and had less variation in women in the US cohort than those in European cohorts. No associations of nitrogen oxide with thyroid function were found. Higher exposures to PM_2.5_ were associated with higher odds of hypothyroxinemia in pregnant women (odds ratio per 5-μg/m^3 ^change, 1.21; 95% CI, 1.00-1.47). Although exposure to PM with an aerodynamic diameter of 10 μm or less was not significantly associated with hypothyroxinemia, the coefficient was similar to that for the association of PM_2.5_ with hypothyroxinemia (odds ratio per 10-μg/m^3 ^change, 1.18; 95% CI, 0.93-1.48). Absorbances of PM_2.5_ and PM with aerodynamic diameter from 2.5 to 10 μg and were not associated with hypothyroxinemia. There was substantial heterogeneity among cohorts with respect to thyroid peroxidase antibodies (*P* for heterogeneity, <.001), showing associations of nitrogen oxide and PM with thyroid autoimmunity only in the women in the Generation R Study.

**Conclusions and Relevance:**

The findings of this study suggest that first-trimester exposures to PM_2.5_ were associated with mild thyroid dysfunction throughout pregnancy. The association of PM_2.5_ exposure with thyroid function during pregnancy is of global health importance because air pollution exposure is widespread and hypothyroxinemia may adversely influence the brain development of offspring.

## Key Points

**Question:**

Is exposure to ambient air pollution in the first trimester associated with thyroid function throughout pregnancy?

**Findings:**

Among 9931 pregnant women in 4 European cohorts and 1 US cohort, an increase of 5 μg/m^3^ in exposure to particulate matter with an aerodynamic diameter of 2.5 μm or less was associated with 20% higher odds of hypothyroxinemia.

**Meaning:**

The findings of this study raise the possibility that exposure to particulate matter might disrupt thyroid function in pregnant women.

## Teaser

This cohort study evaluates the short-term association of exposure to air pollution in the first trimester with thyroid function throughout pregnancy among European and US cohorts.

## Introduction

Exposure to ambient air pollution is a leading contributor to the burden of disease globally. Exposure to air pollutants, such as nitrogen dioxide (NO_2_), nitrogen oxides (NO_x_), particulate matter (PM), and polycyclic aromatic hydrocarbons (PAHs), during pregnancy is associated with brain structural alterations, impaired executive function, learning disabilities, and behavioral problems in offspring.^1,2,3,4^ Oxidative stress, neuroinflammation, and the disruption of the hypothalamus-pituitary-adrenal axis are some underlying factors.^5,6^ Endocrine disruption might be among other mechanisms; for example, PAHs induce activation of the estrogen receptor gene, and PAHs and PM can interfere with nuclear receptors, such as estrogen receptor signaling.^7,8^ Experimental and epidemiological studies have also shown associations between PAH exposure and thyroid function.^9,10^ Nonetheless, evidence on whether exposure to air pollution might also disrupt thyroid signaling and thyroid function is limited.

Thyroid function is of particular importance for pregnant women because of the critical role of thyroid hormones in fetal brain development. Because the fetal thyroid gland achieves its full function only from midgestation on, undetected or inadequately treated thyroid insufficiency in pregnant women adversely influences growth and development of offspring, even in the absence of neonatal hypothyroidism.^11^ Recent evidence suggests that mild thyroid insufficiency, ie, hypothyroxinemia, defined as low free thyroxine (T_4_) levels with normal thyrotropin (TSH) concentrations, during pregnancy may also contribute to impaired cognition and neurodevelopmental disorders in offspring.^12,13,14^ While inadequate iodine intake is a common cause of thyroid insufficiency worldwide, exposure to environmental contaminants is increasingly considered important.^15^ Some studies have examined the association of exposure to ambient and traffic-related air pollution with thyroid function in pregnant women. A 2017 study^16^ of 499 mother-child pairs in Belgium examined the association of third-trimester exposure to PM with an aerodynamic diameter of 2.5 μm or less (PM_2.5_) with maternal and fetal thyroid function. This study found inverse associations of PM_2.5_ exposure with levels of free T_4_, free triiodothyronine, and TSH in fetal cord blood and maternal free T_4_ levels.^16^ In 8077 pregnant women in Shanghai,^17^ exposure to higher concentrations of PM_2.5_ but not NO_2_ in the first and second trimesters was associated with hypothyroxinemia in midgestation. A study in California with 2050 mother-child pairs^18^ showed that newborns who were prenatally exposed to PM_2.5_ and PM with an aerodynamic diameter of 10 μm or less (PM_10_) had higher concentrations of total T_4_ levels in heel-stick blood spots.

A potential association of air pollution exposure with thyroid function during gestation might further clarify underlying mechanisms of hypothyroxinemia in pregnant women. Accordingly, using 5 birth cohorts (4 in Europe and 1 in the United States), we examined whether measures of air pollutants, ie, NO_2_, NO_x_, and PM, averaged in the first trimester were associated with thyroid function and thyroid autoimmunity throughout the pregnancy. We primarily investigated the short-term associations of air pollutants with mild thyroid hormone insufficiency, ie, hypothyroxinemia and high TSH, because of emerging evidence suggesting their implications for fetal development.^12,13,14,19^ We also examined thyroid peroxidase antibody (TPOAb) positivity on the basis of evidence suggesting the association of air pollution exposure with inflammation^20^ and our earlier findings on the association of TPOAb positivity in pregnancy with maternal and offspring health outcomes.^21,22,23^ We specifically focused on air pollution exposure during the first trimester to ensure that air pollution exposure preceded thyroid function measurement.

## Methods

### Participants

This analysis used data from 5 birth cohorts with prenatal recruitment, including the Amsterdam Born Children and Their Development Study (ABCD; Amsterdam, the Netherlands; n = 3867),^24^ the Generation R Study (Rotterdam, the Netherlands; n = 2605),^25^ Infancia y Medio Ambiente (INMA; including the regions of Sabadell, Gipuzkoa, Valencia, and Asturias, Spain; n = 2239),^26^ Rhea (island of Crete, Greece; n = 483),^27^ and Project Viva (eastern Massachusetts, United States; n = 737),^28^ yielding a total sample of 9931. Recruitment periods for the cohorts were as follows: ABCD, January 2003 to March 2004; Generation R, April 2002 to January 2006; INMA, November 2003 to January 2008; Rhea, February 2007 to February 2008; and Project Viva, April 1999 to November 2002.

Iodine status of participants varied among cohorts. Median urinary iodine concentrations of pregnant women in Generation R and Rhea were optimal according to World Health Organization references.^29,30^ In contrast, mild to moderate iodine insufficiency was observed in INMA (except for women in the Gipuzkoa region).^31^ Urinary iodine concentrations were not measured in ABCD or Project Viva. However, reports from pregnant women in Massachusetts showed optimal iodine status,^32^ and iodine status of participants in Amsterdam and Rotterdam are expected to be comparable. There was no pattern suggesting associations of the iodine status of cohorts with air pollution exposure.

In each cohort, we included pregnant women who had data on first-trimester air pollution exposure and gestational thyroid function. For each woman, thyroid assessments were performed once throughout pregnancy, with most measures collected in the first half of pregnancy (ie, gestational age, <17 weeks). We excluded women with twin pregnancies, women with a history of thyroid disease, and women who reported taking medication affecting thyroid function. There were no data available on medication use in INMA. We did not exclude women who tested positive for TPOAb because conditioning on autoimmune processes, an intermediate factor, might introduce bias in the analysis. This study was granted an exception from ethics review and informed consent because we used deidentified data in each cohort. This study followed the Strengthening the Reporting of Observational Studies in Epidemiology (STROBE) reporting guideline.

### Measurements

In ABCD, Generation R, INMA, and Rhea, 3 two-week air pollution monitoring campaigns were performed within 1 year (January 2009 to April 2011). Air pollution concentrations (ie, NO_2_; NO_x_; PM_2.5_ absorbance, determined as the reflectance of PM_2.5_ filters; PM_2.5_; PM_10_; and PM with aerodynamic diameters between 2.5 and 10 μm [PM_2.5-10_]) at the participants’ home addresses were estimated on a daily basis for the whole period of pregnancy using land-use regression following a standardized procedure.^33,34^ Among the INMA regions, data on PM were only available in Sabadell. In Rhea, only PM_10_, PM_2.5_, and PM_2.5-10_ concentrations were estimated. Land-use regression models were developed for each pollutant metric using all measurement sites. We used a back-extrapolation procedure to estimate exposure concentrations for each woman, averaged across the first trimester at the participant’s home address using daily concentrations from routine background monitoring network sites. Land-use regression models explained a large fraction of the spatial variance in measured annual average air pollutant concentrations.^33,34^ In Project Viva, validated prediction models were used to obtain spatially and temporally resolved estimates of daily PM_2.5_ exposure at each participant’s residential address, following a method described elsewhere.^35^ Briefly, this method combined the satellite aerosol optical depth data at a 10 km × 10 km spatial grid with the spatiotemporal land-use regression models based on monitored ground PM_2.5_ measurements. Satellite remote sensing provides an important tool for monitoring aerosols when surface monitors are not available. For measurement of NO_2_ in Project Viva, we calculated hourly ambient concentrations of NO_2_ by averaging data from the Massachusetts Department of Environmental Protection’s greater Boston monitoring sites,^36,37^ then calculated daily and first-trimester NO_2_ exposure. In ABCD, Generation R, INMA, and Project Viva, if more than 1 address was collected during the first trimester, we calculated the weighted average concentrations of all addresses according to the time spent at each address.

Serum concentrations of free T_4_ and TSH were measured at the median gestational age of 13 weeks in ABCD (range, 5-37 weeks), Generation R (range, 6-18 weeks), INMA (range, 7-33 weeks) and Rhea (range, 6-27 weeks). In Project Viva, TSH, total T_4_, and triiodothyronine resin uptake were measured at the median gestational age of 10 weeks (range, 6-21 weeks), allowing for the calculation of the free T_4_ index, an estimate of circulating free T_4_ levels from total T_4_ × triiodothyronine uptake. Cohorts used different assays for measurements of thyroid hormones and TPOAb (eTable 1 in the Supplement).

To define *hypothyroxinemia*, we followed the recommendations of the American Thyroid Association guideline, which describes hypothyroxinemia as free T_4_ concentrations in the lower 2.5th to 5th percentile of the population, despite a normal TSH level.^38^ Following this guideline, we calculated population-specific cutoffs in individuals without a history of thyroid disease or thyroid medication use and those who tested negative for TPOAb. In INMA, TPOAb was not measured, and thus, no exclusions were made based on TPOAb results. We defined *high TSH* as concentrations higher than the 95th percentile of the cohort. Cutoffs were population-specific, and corresponding cutoffs in each cohort appear in eTable 2 in the Supplement. We also tested alternative cutoffs for free T_4_ as well as the cutoff of 0.03 to 2.5 mIU/L for a normal TSH level to examine whether any observed associations were independent of cutoff choice. We used the laboratory recommended cutoffs for TPOAb positivity (eTable 1 in the Supplement). Details on measurements of covariates appear in the eMethods in the Supplement.

### Statistical Analysis

We used Spearman correlations to examine the correlations between concentrations of air pollutants. We performed logistic regression models to assess the short-term association of air pollutants (averaged in the first trimester) with hypothyroxinemia, high TSH, and TPOAb positivity in pregnant women throughout pregnancy. First, we explored any indication of nonlinearity in the associations of air pollutants with thyroid parameters using generalized additive models. Results confirmed the linearity of associations and indicated no threshold effect. Next, associations of air pollutants with hypothyroxinemia, high TSH, and TPOAb positivity were examined in each cohort. Cohort-specific effect estimates from regression models were then combined using a random-effects meta-analysis after exploring the heterogeneity in the estimates among cohorts. We assessed heterogeneity in the estimates using the *Q* test and the *I*^2^ statistic. Similar to previous studies, we reported the odds ratios (ORs) of hypothyroxinemia, high TSH, and TPOAb positivity per 10-μg/m^3 ^change in NO_2_ and PM_10_ levels, 20-μg/m^3 ^change for NO_X_ levels, 5-μg/m^3 ^change for PM_2.5_ and PM_2.5-10_ levels, and 10^−5^ × m^−1^ change for PM_2.5_ absorbance based on the distribution of pollutants.^17^

Selection of confounders was a priori and based on the direct acyclic graph of the study question and factors associated with air pollution exposure and thyroid function.^39,40,41^ We did not adjust for season because, to our knowledge, no evidence exists on the association of season of pregnancy with free T_4_ or TSH levels (despite associations with air pollution).^42,43^ Models included information on maternal age at enrollment; education; country of birth; smoking and alcohol intake during pregnancy; parity; prepregnancy body mass index, calculated as weight in kilograms divided by height in meters squared; gestational age at thyroid measurement; socioeconomic status, defined using information on household income or occupation obtained from self-administered questionnaires; and marital status. We adjusted the analyses in INMA for 4 regions.

Pregnant women who were included in the analysis were different from those excluded because of missing data on exposure and outcome (eTable 3 in the Supplement). To address the selective nonresponse arising from these differences, we used inverse probability weighting. Briefly, we used the available information for eligible women (eTable 4 in the Supplement) to estimate the probability of participation in the study and used the inverse of those probabilities as weights in the analyses so that the results would be representative of the full cohort.

Among participants with exposure and outcome data, information on covariates was missing in less than 10% of participants, except for smoking during pregnancy (266 of 2605 [10.2%] in Generation R), any alcohol intake during pregnancy (76 of 483 [15.7%] in Rhea), socioeconomic status (626 of 3867 [16.2%] in ABCD, 651 of 2605 [25.0%] in Generation R, and 146 of 483 [30.2%] in Rhea), and prepregnancy body mass index (458 of 2605 [17.6%] in Generation R). We addressed missing data in covariates by imputing data using the Stata ice command for chained equations imputation. We created 25 data sets with complete observations, in which analyses were performed using standard combination rules for multiple imputations (eTable 5 in the Supplement).

In a sensitivity analysis, we ran the meta-analysis for hypothyroxinemia excluding Project Viva because a measure of free T_4_ index instead of free T_4_ level was included in that cohort. Additionally, in Project Viva, NO_2_ was measured using central monitors; therefore, we also reran the NO_2_ meta-analysis excluding Project Viva.

All analyses were performed in Stata statistical software version 14.0 (StataCorp) between January 2018 and April 2019. All statistical tests were 2-sided with a significance threshold of *P* < .05.

## Results

**Figure 1.**
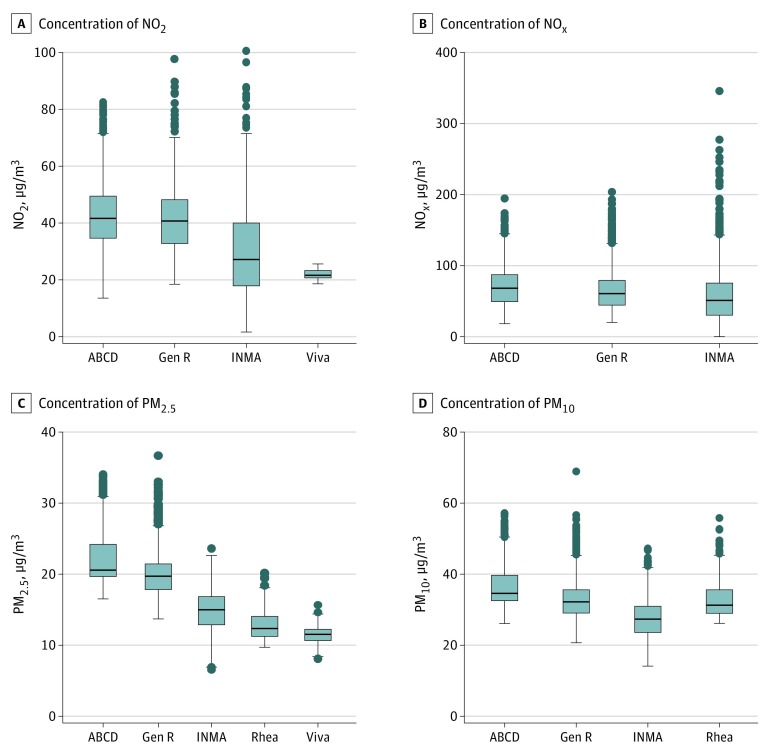
Distribution of Air Pollutants Averaged Across the First Trimester of Pregnancy Data on particulate matter (PM) were only available in the Sabadell subcohort of Infancia y Medio Ambiente (INMA). Center line indicates 50th percentile; upper and lower boundaries of boxes, 75th and 25th percentile, respectively; upper whisker, 75th percentile plus 1.5 times the interquartile range; lower whisker, 25th percentile minus 1.5 times the interquartile range; and circles, any value above or below the whiskers. ABCD indicates the Amsterdam Born Children and Their Development Study; Gen R, the Generation R Study; NO_2_, nitrogen dioxide; NO_x_, nitrogen oxides; PM_2.5_, particulate matter less than 2.5 μm; PM_10_, particulate matter less than 10 μm; and Viva, Project Viva.

Table 1 summarizes participant characteristics. Of 9931 women in the analysis, the mean (SD) age of participants at enrollment was 31.2 (4.8) years, 4853 (48.9%) had more than a secondary education, 5616 (56.6%) were nulliparous, and 584 (5.8%) were single. Overall, 7568 (76.2%) did not smoke during pregnancy, and 2911 (29.3%) reported alcohol intake in pregnancy. A smaller proportion of participants in Project Viva had a low socioeconomic status compared with women in other cohorts (10 [1.4%] in Project Viva, 464 [12.0%] in ABCD, 102 [3.9%] in Generation R, 1189 [53.1%] in INMA, and 40 [8.3%] in Rhea). Women in INMA and Rhea had lower educational levels compared with women in other cohorts (no more than elementary education: 544 [24.4%] in INMA, 97 [20.1%] in Rhea, 683 [17.8%] in ABCD, 428 [17.2%] in Generation R, and 9 [1.2%] in Project Viva). In Project Viva, 103 women (13.9%) tested positive for TPOAb, which was modestly higher than in ABCD (213 [5.5%]), Generation R (145 [6.0%]), and Rhea (45 [9.3%]). Median (interquartile range) concentrations of PM_2.5_ were between 11.5 (10.7-12.3) μg/m^3^ in Project Viva and 20.6 (19.7-24.2) μg/m^3^ in ABCD (Figure 1; eTable 6, eFigure 1, and eFigure 2 in the Supplement). The median (interquartile range) concentration of NO_2_ was 21.6 (20.7-23.3) μg/m^3^ in Project Viva and 41.6 (34.7-49.4) μg/m^3^ in ABCD. Concentrations of NO_2_ and PM_2.5_ were lower and had less variation in Project Viva compared with the 4 European cohorts. The correlations between air pollutants in each cohort reflected high correlations between NO_2_ and NO_x_ (correlation coefficients varied between 0.88 and 0.92) and among PM concentrations (correlation coefficients varied between 0.59 and 0.95) (eTable 7 in the Supplement). Overall, 404 women (4.2%) had hypothyroxinemia, and 506 (6.7%) tested positive for TPOAb.

**Table 1.** Participant Characteristics

Characteristic	No. (%)					
	ABCD, the Netherlands (n = 3867)	Generation R, the Netherlands (n = 2605)	INMA, Spain (n = 2239)	Rhea, Greece (n = 483)	Project Viva, United States (n = 737)	Total (N = 9931)
Age at enrollment, mean (SD), y	33.6 (3.9)	30.8 (4.7)	31.4 (4.2)	29.3 (4.9)	32.5 (4.7)	31.2 (4.8)
Educational levels						
Elementary	683 (17.8)	428 (17.2)	544 (24.4)	97 (20.1)	9 (1.2)	1761 (18.0)
Secondary	1059 (27.6)	756 (30.4)	921 (41.2)	256 (53.0)	179 (24.3)	3171 (32.4)
Higher	2098 (54.6)	1307 (52.4)	769 (34.4)	130 (26.9)	549 (74.5)	4853 (49.6)
Nulliparous	2278 (57.4)	1574 (60.7)	1266 (56.6)	192 (40.4)	366 (49.7)	5616 (56.7)
Foreign country of birth	1210 (31.3)	1115 (42.8)	188 (8.4)	43 (8.9)	119 (16.2)	2675 (27.0)
Single	173 (4.5)	271 (10.9)	38 (1.7)	62 (12.8)	40 (5.4)	584 (6.0)
Never smoked during pregnancy	3503 (90.7)	1767 (75.5)	1485 (68.2)	302 (66.7)	511 (69.5)	7568 (79.1)
Drank alcohol during pregnancy	932 (24.1)	1130 (46.7)	206 (9.6)	102 (25.1)	541 (73.6)	2911 (30.4)
Low socioeconomic status	464 (12.0)	102 (3.9)	1189 (53.1)	40 (8.3)	10 (1.4)	1805 (20.0)
Prepregnancy BMI, mean (SD)	21.9 (3.5)	22.6 (4.4)	22.5 (4.4)	23.4 (5.3)	23.5 (5.3)	23.4 (4.2)
Thyroid function in pregnancy						
Free T_4_, median (IQR), ng/dL	0.7 (0.7-0.8)	1.2 (1.0-1.3)	0.8 (0.7-0.8)	1.2 (1.1-1.3)	2.1 (1.9-2.3)^a^	0.8 (0.7-1.1)^b^
TSH, median (IQR), mIU/L	1.2 (0.8-1.7)	1.7 (1.0-2.5)	1.3 (0.8-1.8)	1.1 (0.7-1.6)	1.2 (0.7-1.9)	1.3 (0.8-1.9)
Hypothyroxinemia^c^	158 (4.1)	112 (4.4)	88 (4.2)	23 (4.8)	23 (3.2)	404 (4.2)
TPOAb positive	213 (5.5)	145 (6.0)	NA	45 (9.3)	103 (13.9)	506 (6.7)
Gestational age at thyroid measurement, median (IQR), wk	13.0 (11.9-14.0)	13.1 (12.1-16.8)	13.0 (12.3-14.0)	13.0 (12.0-15.0)	9.6 (8.7-10.7)	12.9 (11.9-12.3)

Abbreviations: ABCD, Amsterdam Born Children and Their Development; BMI, body mass index (calculated as weight in kilograms divided by height in meters squared); INMA, Infacia y Medio Ambiente; IQR, interquartile range; NA, not available; TPOAb, thyroid peroxidase antibodies; TSH, thyrotropin; T_4_, thyroxine.

SI conversion factor: To convert free T_4_ to pmol/L, multiply by 12.871.

^a^ Calculated from total T_4_ × triiodothyronine resin uptake.

^b^ Excluding Project Viva.

^c^ Defined as free T_4_ below the fifth percentile of cohort distribution despite normal TSH level.

**Figure 2.**
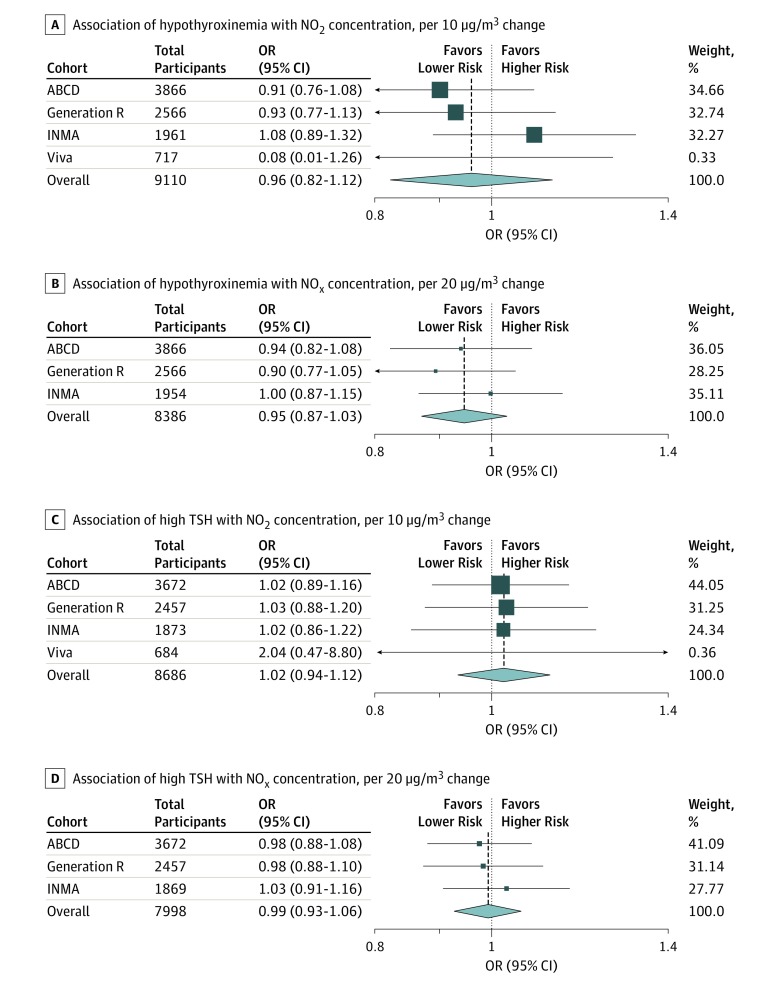
Association of Exposure to Nitrogen Dioxide (NO_2_) and Nitrogen Oxides (NO_x_) in the First Trimester With Thyroid Function During Pregnancy Odds ratios (ORs) were estimated using random-effects meta-analysis by cohort (the Amsterdam Born Children and Their Development Study [ABCD], Generation R, Infancia y Medio Ambiente [INMA], Rhea, and Project Viva [Viva]). Models were adjusted for pregnant maternal age at enrollment, educational level, country of birth, gestational age at thyroid measurement, smoking and alcohol intake during pregnancy, socioeconomic status, marital status, parity, and prepregnancy body mass index (calculated as weight in kilograms divided by height in meters squared). In addition, analysis in INMA was adjusted for region (Sabadell, Gipuzkoa, Valencia, and Asturias). Data are presented as available in each cohort. Hypothyroxinemia was defined as free thyroxine below the fifth percentile of cohort distribution despite normal thyrotropin (TSH) levels. High TSH concentration was defined as levels higher than the 95th percentile. Size of box indicates weight.

The short-term associations of air pollutants (concentrations averaged in the first trimester) with hypothyroxinemia and high TSH throughout pregnancy are presented in Table 2 and Figure 2. Unadjusted analyses appear in eTable 8 in the Supplement, and complete-case analysis without imputation of covariates appears in eTable 9 in the Supplement. We found no associations of NO_2_ and NO_x_ concentrations with hypothyroxinemia during pregnancy in the meta-analysis of estimates in cohorts with available data. When we ran the meta-analysis excluding Project Viva, results remained unchanged (data not shown). Exposures to NO_2_ and NO_x_ were not associated with high TSH during pregnancy.

**Table 2.** Associations of Exposure to Ambient Air Pollutants in the First Trimester With Thyroid Function During Pregnancy

Exposure	Hypothyroxinemia				High TSH			
	Cohorts Included, No.^a^	OR (95% CI)^b^	*P*_h_	*I*^2^, %	Cohorts Included, No.^a^	OR (95% CI)^b^	*P*_h_	*I*^2^, %
NO_2_, per 10-μg/m^3^ change	4	0.96 (0.82-1.12)^c^	.16	41.25	4	1.02 (0.94-1.12)	.83	0
NO_X_, per 20-μg/m^3^ change	3	0.95 (0.87-1.03)	.60	0	3	0.99 (0.93-1.06)	.80	0
PM_2.5_, per 5-μg/m^3^ change	5	1.21 (1.00-1.47)^d^	.37	6.65	5	1.14 (0.88-1.48)	.12	45.58
PM_10_, per 10-μg/m^3^ change	4	1.18 (0.93-1.48)	.33	13.02	4	1.17 (0.87-1.58)	.09	53.72
PM_2.5-10_, per 5-μg/m^3^ change	4	1.05 (0.76-1.45)	.17	40.63	4	1.18 (0.88-1.57)	.12	47.79
PM_2.5_ absorbance, per 10^−5^ × m^−1^ change	4	1.05 (0.88-1.26)	.40	0	4	1.10 (0.95-1.26)	.84	0

Abbreviations: NO_2_, nitrogen dioxide; NO_x_, nitrogen oxides; OR, odds ratio; P_h_, *P* value of heterogeneity; PM_2.5-10_, particulate matter between 2.5 and 10 μm; PM_10_, particulate matter less than 10 μm; PM_2.5_, particulate matter less than 2.5 μm; TSH, thyrotropin.

^a^ Data on PM was only available in Sabadell region of Infacia y Medio Ambiente.

^b^ Estimated using random-effects meta-analysis by cohort (Amsterdam Born Children and Their Development, Generation R, Infacia y Medio Ambiente, Rhea, and Project Viva). The median gestational age at thyroid measurement was at 13 weeks in all cohorts, except for Project Viva, in which measurement was at median gestational age of 10 weeks. Models were adjusted for maternal age at enrollment, educational level, country of birth, gestational age at thyroid measurement, smoking and alcohol intake during pregnancy, socioeconomic status, marital status, parity, and prepregnancy body mass index (calculated as weight in kilograms divided by height in meters squared). Analysis in Infacia y Medio Ambiente was adjusted for region (ie, Sabadell, Gipuzkoa, Valencia, or Asturias).

^c^ Excluding participants of Project Viva: OR per 10-μg/m^3^ change of NO_2_ level, 0.97; 95% CI, 0.87-1.08.

^d^ Excluding participants of Project Viva: OR per 5-μg/m^3^ change of PM_2.5_ level, 1.23; 95% CI, 1.04-1.47.

**Figure 3.**
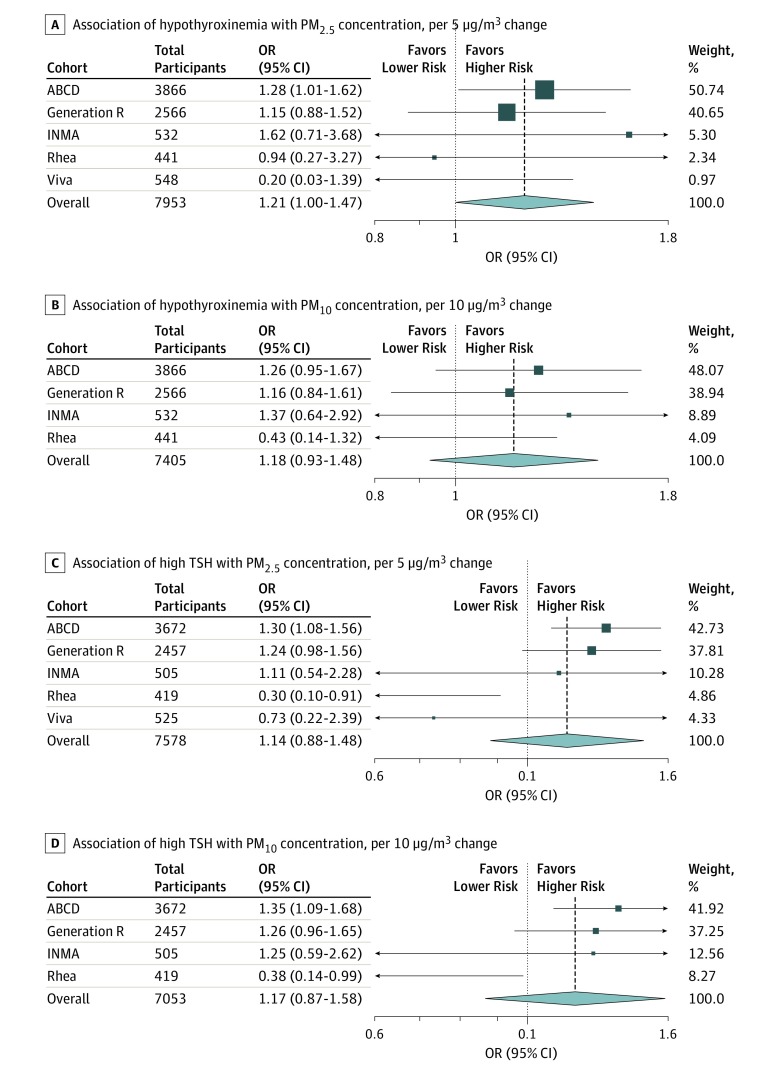
Association of Exposure to Particulate Matter With an Aerodynamic Diameter of 2.5 μm or Less (PM_2.5_) and Particulate Matter With an Aerodynamic Diameter of 10 μm or Less (PM_10_) in the First Trimester With Thyroid Function During Pregnancy Odds ratios (ORs) were estimated using random-effects meta-analysis by cohort (the Amsterdam Born Children and Their Development Study [ABCD], Generation R, Infancia y Medio Ambiente [INMA], Rhea, and Project Viva [Viva]). Models were adjusted for pregnant maternal age at enrollment, educational level, country of birth, gestational age at thyroid measurement, smoking and alcohol intake during pregnancy, socioeconomic status, marital status, parity, and prepregnancy body mass index (calculated as weight in kilograms divided by height in meters squared). Data on particulate matter (PM) was only available in the Sabadell region of INMA. Data are presented as available in each cohort. Hypothyroxinemia was defined as free thyroxine below the fifth percentile of cohort distribution despite normal thyrotropin (TSH) levels. High TSH concentration was defined as levels higher than the 95th percentile. Size of box indicates weight.

Women with higher exposures to PM_2.5_ in the first trimester had higher odds of hypothyroxinemia during pregnancy (OR, 1.21; 95% CI, 1.00-1.47) (Table 2 and Figure 3; eTable 8 and eTable 9 in the Supplement). Exposure to PM_10_ was not associated with higher odds of hypothyroxinemia (OR, 1.18; 95% CI, 0.93-1.48). When we used the 2.5th percentile as the cutoff for free T_4_ level to define hypothyroxinemia, the results did not change (data not shown). The associations of PM exposures and high TSH (concentrations >95th percentile) during pregnancy were not significant (Table 2), but when we used the clinical cutoff of 0.03 to 2.5 mIU/l for a normal TSH level, we found significant associations of exposures to PM_2.5_ and PM_10_ with high TSH (OR per 5-μg/m^3^ change of PM_2.5_ level, 1.23; 95% CI, 1.09-1.39; OR per 10-μg/m^3^ change of PM_10_ level, 1.24; 95% CI, 1.02-1.51). Levels of PM_2.5-10_ and PM_2.5_ absorbance were not associated with hypothyroxinemia (PM_2.5-10_ level: OR, 1.05; 95% CI, 0.76-1.45; PM_2.5_ absorbance level: OR, 1.05; 95% CI, 0.88-1.26) or high TSH (PM_2.5-10_ level: OR, 1.18; 95% CI, 0.88-1.57; PM_2.5_ absorbance level: OR, 1.10; 95% CI, 0.95-1.26), with effect estimates close to null (Table 2). Examination of the association of air pollution exposure with TPOAb positivity showed large heterogeneity among cohorts (*P* values for heterogeneity for NO_2_, PM_10,_ PM_2.5_, and PM_2.5-10_, <.001; for NO_x_, *P* = .01). Therefore, we only performed cohort-specific analysis for TPOAb. In Generation R, higher concentrations of air pollutants were associated with TPOAb positivity (OR per 10-μg/m^3 ^change of NO_2_ level, 1.22; 95% CI, 1.11-1.34; OR per 5-μg/m^3^ change of PM_2.5_ level, 1.76; 95% CI, 1.51-2.04; OR per 10-μg/m^3^ change of PM_10_ level, 1.96; 95% CI, 1.64-2.35). There was no association of air pollution exposure with TPOAb positivity in the other cohorts with available data.

## Discussion

In a large sample from 5 cohorts in Europe and the United States, we found that first-trimester exposures to PM_2.5_ were associated with mild thyroid dysfunction throughout pregnancy. Exposures to NO_x_ and NO_2_ were not associated with hypothyroxinemia or high TSH during pregnancy. In the Generation R cohort, we observed that pregnant women with higher exposures to NO and PM were more likely to be TPOAb positive.

Studies have found associations of PAH exposure with thyroid dysfunction in nonpregnant populations^44^ and of cigarette smoking with thyroid dysfunction in pregnant women.^41^ Three observational studies^16,17,18^ have specifically examined the association of air pollutants with the thyroid function of pregnant women and their neonates. Howe et al^18^ showed that prenatal exposures to PM_2.5_ and PM_10_ but not NO_2_ and ozone were associated with higher neonatal total T_4_. Zhao et al^17^ reported positive associations between residential PM_2.5_ concentrations and maternal hypothyroxinemia during midgestation. Janssen et al^16^ found that third trimester PM_2.5_ exposure was negatively associated with free T_4_ in maternal serum. Our results extend these observations and showed that the association of PM_2.5_ concentrations with hypothyroxinemia were present in the first trimester, the period when the fetus is most sensitive to maternal thyroid dysfunction. In addition, we found no associations of NO_2_ and NO_x_ exposures with thyroid function in pregnancy, similar to a previous study in China.^17^ Consistent findings on null associations of NO_2_ with thyroid function and our findings on null associations of NO_x_ and thyroid function, combined with observed associations of PM with thyroid function, suggest that the association of air pollutants with thyroid function may be mostly associated with PM. Importantly, our findings also confirm that the associations of air pollution exposure with thyroid dysfunction in pregnant women are present with concentrations of pollutants at levels much lower that the study in China,^17^ as shown in Belgium and California.^16,18^

While our findings indicate a short-term association of PM exposure with thyroid function, the mechanisms of this association are not fully understood and need further investigation. Although speculative, direct interference in the intracellular action of deiodinase enzymes and the induction of oxidative stress and inflammation are likely among the short-term mechanisms.^5,45^ Autoimmune processes might act on thyroid function in a longer period. In women of reproductive age, autoimmunity is a common cause of thyroid dysfunction in iodine-sufficient areas. Earlier studies among pregnant women have shown that prolonged lead exposure is associated with TPOAb positivity and subsequently low thyroid function.^46^ We found that exposure to PM during early pregnancy was associated with higher odds of thyroid autoimmunity in Generation R, an iodine-sufficient cohort in the Netherlands. The observed associations between PM exposures and low thyroid function and the null association with NO_2_, NO_x_, and PM_2.5_ absorbance, a measure of black carbon, suggest that the composition of PM rather than the general markers of traffic-related pollution may be responsible for thyroid disruption. One hypothesis—supported by in vitro studies^47^—is that PAHs are associated with the thyroid toxic effects of PM exposure, but we cannot rule out the role of other components, such as trace elements.

The cohorts in our study varied with respect to the concentrations of air pollution exposure. For example, ABCD and Generation R had higher concentrations of PM_2.5_ exposure compared with other cohorts. Exposure to concentrations of NO_2_ and PM measures in the European cohorts were positive and moderate to strong, but there was a negative and small correlation between NO_2_ and PM_2.5_ in the US cohort Project Viva. These differences can be explained by varying sources for exposure to pollutants in different regions as well as different exposure assessment methods in European cohorts and in Project Viva. Nonetheless, examination of the heterogeneity in estimates for analysis of the association of NO_2_ and PM_2.5_ with thyroid function confirmed that estimates among cohorts could be combined in the meta-analysis. There was an inverse but imprecise association of PM_2.5_ with hypothyroxinemia in Project Viva, the smallest cohort with the lowest concentrations of PM_2.5_, suggesting that the association might be present at a threshold of exposure. Nonetheless, results of analyses using generalized additive models to create the smoothing curve spline confirmed no threshold effect of pollutants. With regard to TPOAb positivity, appropriate testing showed large heterogeneity among cohorts. In particular, a larger number of women were positive for TPOAb in Project Viva compared with other cohorts, potentially explained by natural variation, use of different assays, slightly higher mean age in Project Viva at the time of assessment, and earlier measurement during pregnancy. Also, the association of air pollution exposure with TPOAb positivity was only present in Generation R. Further investigation is needed to determine whether the iodine status of the cohort population or other characteristics might explain the differences in the association of air pollution exposure with thyroid autoimmunity.

### Strengths and Limitations

This study has several strengths, including a large number of participants from regions with different iodine status and diverse sociodemographic characteristics in Europe and the United States. Nonetheless, this study had important limitations. We had only measures of TSH, free T_4_, and TPOAb during pregnancy (mostly in the first half of pregnancy), and measurements were performed using different assays among cohorts. While the absolute concentrations of TSH and free T_4_ can vary between assays, we defined the outcomes by population- and assay-specific cutoffs to overcome any issues related to the interchangeability of absolute-concentration assay results. We adjusted the models for history of smoking in pregnancy, but we did not have data on secondhand tobacco smoke exposure in all cohorts. We relied on residential addresses to estimate air pollution exposure, without consideration for within-individual spatial variation in exposure. Furthermore, estimation of air pollution exposure in European cohorts did not account for temporal variation during the first trimester, and estimation of NO_2_ exposure in Project Viva did not account for spatial variation between individuals. Another limitation concerning the European cohorts is that air pollution exposure models were developed based on monitoring campaigns performed between 2009 and 2011 and used to estimate exposures of pregnant women in preceding years. We used routine monitoring data to back-extrapolate the concentrations to the exact first trimester of pregnancy. Therefore, we assumed that the spatial distribution of the sources and predictors of air pollution levels remained stable over time, as previous research has shown.^36^ We did not have history of addresses in pregnant women and thus could not examine the association of air pollution exposure prior to pregnancy with thyroid function. Subsequently, no conclusion on the critical window of exposure or accumulation of risk can be drawn from this analysis.

## Conclusions

The findings of this study suggest that first-trimester exposures to PM_2.5_ were associated with mild thyroid dysfunction throughout pregnancy. The association of PM_2.5_ exposure with thyroid function in pregnant women is of global health importance because air pollution exposure is widespread and hypothyroxinemia may adversely influence offspring brain development.

## Notes

Supplement. **eMethods. **Measurements of Covariates **eTable 1. **Immunoassays Used for Measurement of Thyroid Parameters in 5 Participating Cohorts **eTable 2. **Cohort-Specific Cutoffs for Free Thyroxine (T_4_) and Thyrotropin (TSH) in 5 Cohorts **eTable 3. **Participants’ Characteristics in Pregnant Women Included in the Analysis and Those Women Excluded Because of Missing Data in Exposure and/or Outcome **eTable 4. **Variables Used in Forward Selection Logistic Regression Model to Calculate Inverse Probability of Attrition Weights **eTable 5. **Imputation Modelling **eTable 6.** Concentrations of Air Pollutants Averaged Over the First Trimester of Pregnancy Based on Residential Addresses in 5 Cohorts **eTable 7. **Spearman Correlations Between Concentrations of Traffic-Related Air Pollutants in 5 Cohorts **eTable 8. **Associations Between Exposure to Ambient Air Pollutants in the First Trimester and Thyroid Function During Pregnancy: Unadjusted Analysis **eTable 9. **Associations Between Exposure to Ambient Air Pollutants in the First Trimester and Thyroid Function During Pregnancy: Complete Case Analysis **eFigure 1. **Distribution of PM_2.5-10_ and PM_2.5_ Absorbance Averaged Across the First Trimester of Pregnancy in 5 Birth Cohorts **eFigure 2. **Exposure to PM_2.5-10_ and PM_2.5_ Absorbance in the First Trimester and Thyroid Function During Pregnancy
