# Supplementary material for: Association of Exposure to Ambient Air Pollution With Thyroid Function During Pregnancy
Source: JAMA Netw Open. 2019 Oct 16;2(10):e1912902. doi: 10.1001/jamanetworkopen.2019.12902 (PMC6806433; doi:10.1001/jamanetworkopen.2019.12902)
Supplement: Supplement. — eMethods. Measurements of Covariates eTable 1. Immunoassays Used for Measurement of Thyroid Parameters in 5 Participating Cohorts eTable 2. Cohort-Specific Cutoffs for Free Thyroxine (T4) and Thyrotropin (TSH) in 5 Cohorts eTable 3. Participants’ Characteristics in Pregnant Women Included in the Analysis and Those Women Excluded Because of Missing Data in Exposure and/or Outcome eTable 4. Variables Used in Forward Selection Logistic Regression Model to Calculate Inverse Probability of Attrition Weights eTable 5. Imputation Modelling eTable 6. Concentrations of Air Pollutants Averaged Over the First Trimester of Pregnancy Based on Residential Addresses in 5 Cohorts eTable 7. Spearman Correlations Between Concentrations of Traffic-Related Air Pollutants in 5 Cohorts eTable 8. Associations Between Exposure to Ambient Air Pollutants in the First Trimester and Thyroid Function During Pregnancy: Unadjusted Analysis eTable 9. Associations Between Exposure to Ambient Air Pollutants in the First Trimester and Thyroid Function During Pregnancy: Complete Case Analysis eFigure 1. Distribution of PM2.5-10 and PM2.5 Absorbance Averaged Across the First Trimester of Pregnancy in 5 Birth Cohorts eFigure 2. Exposure to PM2.5-10 and PM2.5 Absorbance in the First Trimester and Thyroid Function During Pregnancy [file jamanetwopen-2-e1912902-s001.pdf]

Supplementary Online Content

Ghassabian A, Pierotti L, Basterrechea M, et al. Association of exposure to ambient air pollution with thyroid function during pregnancy. *JAMA Netw Open.* 2019;2(10):e1912902. doi:10.1001/jamanetworkopen.2019.12902

eMethods. Measurements of Covariates

eTable 1. Immunoassays Used for Measurement of Thyroid Parameters in 5 Participating Cohorts

eTable 2. Cohort-Specific Cutoffs for Free Thyroxine (T_4) and Thyrotropin (TSH) in 5 Cohorts

eTable 3. Participants' Characteristics in Pregnant Women Included in the Analysis and Those Women Excluded Because of Missing Data in Exposure and/or Outcome

eTable 4. Variables Used in Forward Selection Logistic Regression Model to Calculate Inverse Probability of Attrition Weights

eTable 5. Imputation Modelling

eTable 6. Concentrations of Air Pollutants Averaged Over the First Trimester of Pregnancy Based on Residential Addresses in 5 Cohorts

eTable 7. Spearman Correlations Between Concentrations of Traffic-Related Air Pollutants in 5 Cohorts

eTable 8. Associations Between Exposure to Ambient Air Pollutants in the First Trimester and Thyroid Function During Pregnancy: Unadjusted Analysis

eTable 9. Associations Between Exposure to Ambient Air Pollutants in the First Trimester and Thyroid Function During Pregnancy: Complete Case Analysis

eFigure 1. Distribution of $PM_{2.5-10}$ and $PM_{2.5}$ Absorbance Averaged Across the First Trimester of Pregnancy in 5 Birth Cohorts

eFigure 2. Exposure to $PM_{2.5-10}$ and $PM_{2.5}$ Absorbance in the First Trimester and Thyroid Function During Pregnancy

This supplementary material has been provided by the authors to give readers additional information about their work.

© 2019 Ghassabian A et al. *JAMA Network Open.*

eMethods. Measurements of Covariates

Information on participants' age at enrollment (year), country of birth (country of the cohort, foreign country), marital status (single, yes/no), tobacco smoke use (never, yes/no), and parity (nulliparous, one, two or more) were collected by self-reported questionnaires or in face-to-face interviews during the first prenatal visit or throughout pregnancy. In ABCD, alcohol intake was asked as the number of glass for alcoholic beverages per week during pregnancy. In Generation R, women were asked during each trimester of pregnancy if they had used any alcoholic drinks during the three months prior to the date of visit. In Rhea, the food-frequency questionnaire (FFQ) was used during the second trimester to assess alcohol intake (gram/day) and then categorized into drinks per week directly. In INMA, weekly alcohol intake was asked by type in categories at enrollment. Project Viva obtained data on consumption of alcoholic beverages from the FFQ that expectant mothers completed in early and mid-pregnancy. Maternal height and weight were measured (Generation R, INMA, and Rhea) or self-reported (ABCD, Project Viva) at enrollment and used to estimate pre-pregnancy body mass index (BMI, kg/m^2). Maternal education was the highest completed schooling. Cohort-specific categories for elementary, secondary, and higher levels of education were: ABCD (<6 years /6–10 years/ >10 years); Generation R (primary/secondary/university degree or higher); Rhea (compulsory education up to 9 years/3-5 additional years/university degree or higher); INMA (<11 years /12-15 years/ ≥16 years of education); and Project Viva (high school or less, some college, and college graduate or graduate degree). Social class was described using information on household income or on occupation obtained from self-administered questionnaires. Social class was classified as low income/social class, medium income/social class, and high income/social class according to the International Standard Classification of Occupations (except for Project Viva in which annual household income was used only). Gestational age at the time of thyroid measurement (week) was based on ultrasound data and, if unavailable, on the first day of last menstrual period.

© 2019 Ghassabian A et al. *JAMA Network Open.*

eTable 1. Immunoassays Used for Measurement of Thyroid Parameters in 5 Participating Cohorts

**Cohort**	**Assay**
ABCD	Thyroid hormones: Access immunoanalyzer of Beckman Coultier, Inc.Thyroid peroxidase antibody: Elisa ELIZEN TG Ab (E-CK-96), Zentech, Luik, Belgium (TPOAb positive if >80 kIU/L). Interassay coefficients of variation for free T4: 3.1-5.0%. Interassay coefficients of variation for TSH: 5.0%. Interassay coefficient variation for TPOAb: 13.4%.
Generation R	Thyroid hormones: chemiluminescence assays (Vitros ECI; Ortho Clinical Diagnostics, Rochester, NY, USA). Thyroid peroxidase antibody: Phadia 250 immunoassay (Phadia AB, Uppsala, Sweden) (TPOAb positive if >60 IU/ml). Interassay and intra-assay coefficients of variation for free T_4: 4.7-5.4% and 1.4-2.7%. Interassay and intra-assay coefficients of variation for TSH: 2.5-4.1% and 1.0-1.2%. Interassay and intra-assay coefficients for TPOAb: 2.5-4.8% and 4.1-4.7%.
INMA	Thyroid hormones: AutoDEL-FIA (PerkinElmer Life and Analytical Sciences, Wallac Oy, Turku, Finland) and a lanthanide metal europium (Eu) label. Interassay and intra-assay coefficients of variation for free T_4: 4.0-6.1% and 3.0-3.7%. Interassay and intra-assay coefficients of variation for TSH: 2.6-3.0% and 1.7-7.7%.
Rhea	Thyroid hormones and thyroid peroxidase antibody: IMMULITE 2000 immunoassay system (Siemens Healthcare Diagnostics, ILL 60015-0778, USA) (TPOAb positive if ≥35 IU/mL). Interassay and intra-assay coefficients of variation <12.5% and < 12.5% for TSH. Interassay and intra-assay coefficients of variation <7.1% and <7.8% for free T4. Interassay and intra-assay coefficients of variation <7.2% and 7.4% for TPOAb.
Project Viva	Thyroid hormones: Chemiluminescence assays (Centaur; Bayer, Fernwald, Germany). Thyroid peroxidase antibody: the Nichols Advantage assay (Nichols Institute Diagnostics) (TPOAb positive if >2.0 IU/ml). Interassay and intra-assay coefficients of variation <4.7% and < 2.9% for TSH.

© 2019 Ghassabian A et al. *JAMA Network Open.*

eTable 2. Cohort-Specific Cutoffs for Free Thyroxine (T_4) and Thyrotropin (TSH) in 5 Cohorts

	Free T_4 (pmol/L)		TSH (mIU/L)	
	5th	95th	5th	95th
ABCD	7.46	12.09	0.26	2.65
Generation R	11.00	20.42	0.25	4.18
INMA	8.35	13.10	0.27	3.05
Rhea	12.16	19.03	0.24	2.60
Project Viva [a]	1.60	2.70	0.13	2.93

[a] In Project Viva, free T_4 index was calculated from total T_4 × triiodothyronine resin uptake

Values were calculated in participants with no thyroid disease or thyroid medication use who were thyroid peroxidase antibody negative.

© 2019 Ghassabian A et al. *JAMA Network Open.*

eTable 3. Participants' Characteristics in Pregnant Women Included in the Analysis and Those Women Excluded Because of Missing Data in Exposure and/or Outcome

	**ABCD (NL)**			**Generation R (NL)**			**INMA (ES)**			**Rhea (GR)**			**Project Viva (US)**		
	**N Incl. n=4399**	**Incl. n=3867**	**p**	**N Incl. n=6004**	**Incl. n=2605**	**p**	**N Incl. n=263**	**Incl. n=2239**	**p**	**N Incl. n=355**	**Incl. n=483**	**p**	**N Incl. n=1298**	**Incl. n=737**	**p**
Age at enrollment, y	33.5 (4.5)	33.2 (4.9)	<0.001	29.1 (5.5)	30.8 (4.7)	<0.001	31.7 (5.2)	31.4 (4.2)	0.47	31.7 (5.2)	31.4 (4.2)	0.20	31.3 (5.4)	35.5 (4.8)	<0.001
Educational levels, %															
Elementary	28.5	17.8	<0.001	31.8	17.2	<0.001	31.6	24.4	<0.001	22.4	20.1	0.22	4.1	1.2	<0.001
Secondary	28.0	27.6		31.3	30.4		46.2	41.2		46.9	53.0		36.9	24.3	
Higher	43.5	54.6		36.9	52.4		22.2	34.4		30.7	26.9		59.0	74.5	
Nulliparous, %	55.7	60.5	0.33	53.6	60.7	<0.001	55.4	56.6	<0.001	43.6	40.4	0.57	46.8	49.7	0.46
Country of birth, foreign, %	45.3	31.3	<0.001	58.7	42.8	<0.001	12.4	8.4	0.04	11.9	8.9	0.16	24.2	16.2	<0.001
Marital status, single, %	9.3	7.1	<0.001	16.7	11.0	<0.001	1.8	1.7	0.79	10.5	13.0	0.29	10.7	5.4	<0.001
History of smoking, never, %	92.2	93.8	0.13	71.3	75.6	<0.001	60.4	68.2	0.01	66.2	66.7	0.57	68.1	69.5	0.05
Alcohol intake in pregnancy, %	17.1	24.1	<0.001	35.5	46.7	<0.001	12.3	9.6	0.27	34.7	25.1	0.01	60.2	73.6	<0.001
Social class, low, %	16.1	12.0	<0.001	5.2	3.4	<0.001	54.4	53.1	0.54	6.4	8.3	0.17	6.8	1.4	<0.001
Pre-pregnancy BMI, kg/m^2	22.8 (3.7)	22.6 (3.4)	0.30	23.7 (4.4)	23.5 (4.2)	0.11	23.7 (4.1)	23.5 (4.2)	0.39	23.7 (4.1)	23.5 (4.2)	0.66	25.0 (5.7)	24.5 (5.1)	0.03

BMI: Body mass index; Incl.: Included in the analysis; N Inc.: Not included in the analysis because of missing data in exposure and outcome.

Numbers are mean (SD) for continuous variables (age and pre-pregnancy BMI) and percentages for categorical variables.

© 2019 Ghassabian A et al. *JAMA Network Open.*

eTable 4. Variables Used in Forward Selection Logistic Regression Model to Calculate Inverse Probability of Attrition Weights

	Cohorts									
**Variables**	**ABCD**		**Generation R**		**INMA**		**Rhea**		**Project Viva**	
	Explored	Included	Explored	Included	Explored	Included	Explored	Included	Explored	Included
Age of pregnant women at enrollment	X		X	X	X	X	X	X	X	X
Gestational age at thyroid measurement	X	X	X	X	X	X	X	X	X	
Educational levels	X		X		X	X	X	X	X	X
Marital status	X		X		X	X	X	X	X	
Country of birth	X	X	X	X	X	X	X	X	X	X
History of smoking	X	X	X	X	X		X		X	
Alcohol intake during pregnancy	X		X	X	X	X	X	X	X	X
Socioeconomic status	X		X		X	X	X		X	X
Pre-pregnancy body mass index	X		X		X	X	X		X	
Parity	X		X	X	X	X	X		X	
Second hand smoking	X		X	X	X		X	X	X	
Intelligence Quotient (IQ)			X	X	X		X		X	
Psychiatric symptoms	X		X		X		X		X	
Urinary iodine during pregnancy			X		X		X		X	
Urinary creatinine during pregnancy			X		X		X		X	
Iodine from diet (µg/day)					X	X	X		X	
Gestational age at iodine assessment			X		X	X	X	X	X	

© 2019 Ghassabian A et al. *JAMA Network Open.*

eTable 5. Imputation Modelling

**Software used and key settings:** STATA 14.0 software (Stata Corporation, College Station, Texas) – Ice command (with 10 cycles)
**Number of imputed datasets created:** 25
**Variables included in the imputation procedure for ABCD:**<br>Thyroid stimulating hormone (TSH) in pregnancy (mIU/l), Free thyroxine (T_4) in pregnancy (µg/dl), particulate matter $(PM)_{2.5}$ absorbance - averaged in the first trimester of pregnancy, $PM_{2.5\text{-}10}$ - averaged in the first trimester of pregnancy, NO_2 averaged in the first trimester of pregnancy, pregnant women's characteristics (education levels, country of birth, women´s age at enrollment, gestational age at thyroid measurement, history of smoking, alcohol intake during pregnancy, social class, marital status, parity, pre-pregnancy body mass index, and psychiatric symptoms.<br><br>**Variables included in the imputation procedure for Generation R:**<br>TSH in pregnancy (mIU/l), free T_4 in pregnancy (µg/dl), $PM_{2.5}$ absorbance - averaged in the first trimester of pregnancy, $PM_{2.5\text{-}10}$ - averaged in the first trimester of pregnancy, NO_2 averaged in the first trimester of pregnancy, pregnant women's characteristics (education level, country of birth of parents, women´s age at enrollment, gestational age at thyroid measurement, history of smoking, alcohol intake during pregnancy, social class, marital status, parity, secondhand smoking, IQ, pre-pregnancy body mass index, and psychiatric symptoms, urinary iodine concentrations during pregnancy, urinary creatinine concentrations during pregnancy, and gestational age at iodine assessment.<br><br>**Variables included in the imputation procedure for INMA:**<br>TSH in pregnancy (mIU/l), free T_4 in pregnancy (µg/dl), $PM_{2.5}$ absorbance - averaged in the first trimester of pregnancy, $PM_{2.5\text{-}10}$ - averaged in the first trimester of pregnancy, NO_2 averaged in the first trimester of pregnancy, maternal characteristics (education levels, country of birth, women´s age at enrollment, gestational age at thyroid measurement, history of smoking, alcohol intake during pregnancy, social class, marital status, parity, secondhand smoking, IQ, pre-pregnancy body mass index, psychopathological symptoms, urinary iodine concentrations during pregnancy, urinary creatinine concentrations during pregnancy, iodine from diet (µg/day) and gestational age at iodine assessment (data on urinary iodine concentrations and gestational age at assessment were not available in the region of Asturias; data on PM was only available in Sabadell).<br><br>**Variables included in the imputation procedure for Rhea:**<br>TSH in pregnancy (mIU/l), free T_4 in pregnancy (µg/dl), $PM_{2.5}$ absorbance - averaged in the first trimester of pregnancy, $PM_{2.5\text{-}10}$ - averaged in the first trimester of pregnancy, pregnant women's characteristics (education levels, country of birth, women´s age at enrollment, gestational age at thyroid measurement, history of smoking, alcohol intake during pregnancy, social class, marital status, parity, secondhand smoking, IQ, pre-pregnancy body mass index, psychopathological symptoms, urinary iodine concentrations during pregnancy, urinary creatinine concentrations during pregnancy, iodine from diet (µg/day) and gestational age at iodine assessment<br><br>**Variables included in the imputation procedure for Project Viva:**<br>TSH in pregnancy (mIU/l), free T_4 index in pregnancy, $PM_{2.5}$ - averaged in the first trimester of pregnancy, Black carbon - averaged in the first trimester, NO_2 - averaged in the second trimester, pregnant women's characteristics (education levels, country of birth, women´s age at enrollment, gestational age at thyroid measurement, history of smoking, alcohol intake during pregnancy, social class, marital status, parity, secondhand smoking, IQ, pre-pregnancy body mass index, psychopathological symptoms, urinary iodine concentrations during pregnancy, urinary creatinine concentrations during pregnancy, iodine levels from diet (µg/day), gestational age at iodine assessment
**Treatment of binary/categorical variables:** logistic and multinomial models
**Statistical interactions included in imputation models:** none

© 2019 Ghassabian A et al. *JAMA Network Open.*

eTable 6. Concentrations of Air Pollutants Averaged Over the First Trimester of Pregnancy Based on Residential Addresses in 5 Cohorts

**ABCD**	Median	Interquartile range
NO_2,	41.6	14.7
NO_x	68.7	38.3
$PM_{2.5}$	20.6	4.5
PM_{10}	34.6	7.20
$PM_{2.5-10}$	13.9	3.1
$PM_{2.5}$ absorbance	2.00	1.1
**Generation R**		
NO_2	40.7	15.5
NO_x	60.7	34.8
$PM_{2.5}$	19.7	3.6
PM_{10}	32.2	6.5
$PM_{2.5-10}$	12.0	3.0
$PM_{2.5}$ absorbance	1.7	0.7
**INMA**		
NO_2	27.2	22.2
NO_x	51.3	45.2
$PM_{2.5}$	15.0	4.0
PM_{10}	27.3	7.4
$PM_{2.5-10}$	12.2	3.9
$PM_{2.5}$ absorbance	2.3	1.6
**Rhea**		
$PM_{2.5}$	12.4	2.9
PM_{10}	31.3	6.7
$PM_{2.5-10}$	19.0	3.8
$PM_{2.5}$ absorbance	1.5	0.7
**Project Viva**		
NO_2	21.6	2.6
$PM_{2.5}$	11.5	1.6

Nitrogen dioxide: NO_2; Nitrogen oxides: NO_x; Not available: NA; Particulate matter less than 10µm: PM_{10}; Particulate matter less than 2.5µm: $PM_{2.5}$; Particulate matter between 2.5 and 10µm: PM_{coarse}; Reflectance of $PM_{2.5}$ filters: $PM_{2.5}$ absorbance.

Concentrations are in µg/m^3 except for $PM_{2.5}$ absorbance, which is expressed in $10^{-5}m^{-1}$. Data on PM was only available in Sabadell region of INMA

© 2019 Ghassabian A et al. *JAMA Network Open.*

eTable 7. Spearman Correlations Between Concentrations of Traffic-Related Air Pollutants in 5 Cohorts

**ABCD**	NO_2	NO_x	PM_{10}	$PM_{2.5}$	$PM_{2.5\text{-}10}$
NO_x	0.912				
PM_{10}	0.427	0.287			
$PM_{2.5}$	0.314	0.205	0.948		
$PM_{2.5\text{-}10}$	0.496	0.330	0.951	0.819	
$PM_{2.5}$ absorbance	0.868	0.868	0.543	0.498	0.534
**Generation R**					
NO_x	0.925				
PM_{10}	0.443	0.378			
$PM_{2.5}$	0.347	0.241	0.954		
$PM_{2.5\text{-}10}$	0.438	0.380	0.875	0.838	
$PM_{2.5}$ absorbance	0.924	0.912	0.517	0.415	0.457
**INMA**					
NO_x	0.880				
PM_{10}	0.839	0.862			
$PM_{2.5}$	0.796	0.837	0.932		
$PM_{2.5\text{-}10}$	0.780	0.794	0.941	0.766	
$PM_{2.5}$ absorbance	0.793	0.970	0.864	0.868	0.769
**Rhea**					
$PM_{2.5}$	NA	NA	0.822		
$PM_{2.5\text{-}10}$	NA	NA	0.936	0.592	
$PM_{2.5}$ absorbance	NA	NA	0.101	0.202	-0.030
**Project Viva**					
$PM_{2.5}$	-0.198	NA	NA	NA	NA

Nitrogen dioxide: NO_2; Nitrogen oxides: NO_x; Not available: NA; Particulate matter less than 10µm: PM_{10}; Particulate matter less than 2.5µm: $PM_{2.5}$; Particulate matter between 2.5 and 10µm: $PM_{2.5\text{-}10}$; Reflectance of $PM_{2.5}$ filters: $PM_{2.5}$ absorbance.

Data on PM was only available in Sabadell region of INMA.

© 2019 Ghassabian A et al. *JAMA Network Open.*

eTable 8. Associations Between Exposure to Ambient Air Pollutants in the First Trimester and Thyroid Function During Pregnancy: Unadjusted Analysis

	Hypothyroxinemia					High TSH			
	**N[a]**	**OR (95%CI)**	**P_h**	**I^2**		**N[a]**	**OR (95%CI)**	**P_h**	**I^2**
NO_2 (per Δ10 μg/m^3)	4	0.88 (0.69-1.12)	0.01	75.00		4	1.02 (0.94-1.12)	0.78	0.00
NO_X (per Δ 20 μg/m^3)	3	0.91 (0.83-1.00)	0.25	27.74		3	1.00 (0.94-1.06)	0.79	0.00
$PM_{2.5}$ (per Δ 5 μg/m^3)	5	1.11 (0.89-1.39)	0.28	21.48		5	1.16 (0.94-1.45)	0.19	34.39
PM_{10} (per Δ 10 μg/m^3)	4	1.02 (0.79-1.31)	0.27	23.68		4	1.16 (0.87-1.55)	0.09	53.67
$PM_{2.5-10}$ (per Δ 5 μg/m^3)	4	0.90 (0.60-1.37)	0.03	66.02		4	1.12 (0.84-1.50)	0.10	52.34
$PM_{2.5}$absorbance (per Δ $10^{-5}m^{-1}$)	4	0.97 (0.82-1.14)	0.52	0.00		4	1.10 (0.96-1.26)	0.90	0.00

CI: Confidence interval; OR: Odds ratio; NO_2: Nitrogen dioxide; NO_x: Nitrogen oxides; P_h: P value of heterogeneity; PM_{10}: Particulate matter less than 10μm; $PM_{2.5}$: Particulate matter less than 2.5μm; $PM_{2.5-10}$: Particulate matter between 2.5 and 10μm; $PM_{2.5}$ absorbance: Reflectance of $PM_{2.5}$ filters; T_4: Thyroxine; TPOAb: Thyroid peroxidase antibodies; TSH: Thyroid stimulating hormone.

Odds ratios (95%CI) were estimated using random-effects meta-analysis by cohort (ABCD, Generation R, INMA, Rhea, and Project Viva).

We defined hypothyroxinemia as free T_4 below the 5th percentile of cohort distribution despite normal TSH. Higher TSH was defined as values > 95th percentile. I^2 refers to the percentage of the total variability due to between-areas heterogeneity. P value of heterogeneity was estimated using the Cochran's Q test.

[a] Number of cohorts included in the meta-analysis. Data on PM was only available in Sabadell region of INMA.

© 2019 Ghassabian A et al. *JAMA Network Open.*

eTable 9. Associations Between Exposure to Ambient Air Pollutants in the First Trimester and Thyroid Function During Pregnancy: Complete Case Analysis

	Hypothyroxinemia					High TSH			
	N[a]	OR (95%CI)	P_h	I^2		N[a]	OR (95%CI)	P_h	I^2
NO_2 (per Δ10 μg/m^3)	4	0.97 (0.83-1.12)	0.30	19.30		4	1.01 (0.92-1.11)	0.65	0.00
NO_X (per Δ 20 μg/m^3)	3	0.95 (0.86-1.04)	0.64	0.00		3	0.98 (0.92-1.05)	0.69	0.00
$PM_{2.5}$ (per Δ 5 μg/m^3)	5	1.28 (0.99-1.66)	0.69	0.00		5	1.09 (0.71-1.67)	0.03	65.90
PM_{10} (per Δ 10 μg/m^3)	4	1.30 (1.01-1.67)	0.36	8.1		4	1.11 (0.81-1.53)	0.09	50.60
$PM_{2.5-10}$ (per Δ 5 μg/m^3)	4	1.12 (0.84-1.49)	0.56	0.00		4	1.11 (0.74-1.65)	0.07	58.00
$PM_{2.5}$absorbance (per Δ $10^{-5}m^{-1}$)	4	1.03 (0.83-1.28)	0.88	0.00		4	1.06 (0.91-1.24)	0.59	0.00

CI: Confidence interval; OR: Odds ratio; NO_2: Nitrogen dioxide; NO_x: Nitrogen oxides; P_h: P value of heterogeneity; PM_{10}: Particulate matter less than 10μm; $PM_{2.5}$: Particulate matter less than 2.5μm; $PM_{2.5-10}$: Particulate matter between 2.5 and 10μm; $PM_{2.5}$ absorbance: Reflectance of $PM_{2.5}$ filters; T_4: Thyroxine; TPOAb: Thyroid peroxidase antibodies; TSH: Thyroid stimulating hormone.

Odds ratios (95%CI) were estimated using random-effects meta-analysis by cohort (ABCD, Generation R, INMA, Rhea, and Project Viva).

We defined hypothyroxinemia as free T_4 below the 5th percentile of cohort distribution despite normal TSH. Higher TSH was defined as values > 95th percentile. I^2 refers to the percentage of the total variability due to between-areas heterogeneity. P value of heterogeneity was estimated using the Cochran's Q test.

Models were adjusted for pregnant women's age at enrollment, educational level, country of birth, gestational age at thyroid measurement, history of smoking, alcohol intake during pregnancy, socioeconomic status, marital status, parity, and pre-pregnancy body mass index. In addition, analysis in INMA was adjusted for regions (Sabadell, Gipuzkoa, Valencia, and Asturias).

[a] Number of cohorts included in the meta-analysis. Data on PM was only available in Sabadell region of INMA.

© 2019 Ghassabian A et al. *JAMA Network Open.*

eFigure 1. Distribution of $PM_{2.5-10}$ and $PM_{2.5}$ Absorbance Averaged Across the First Trimester of Pregnancy in 5 Birth Cohorts

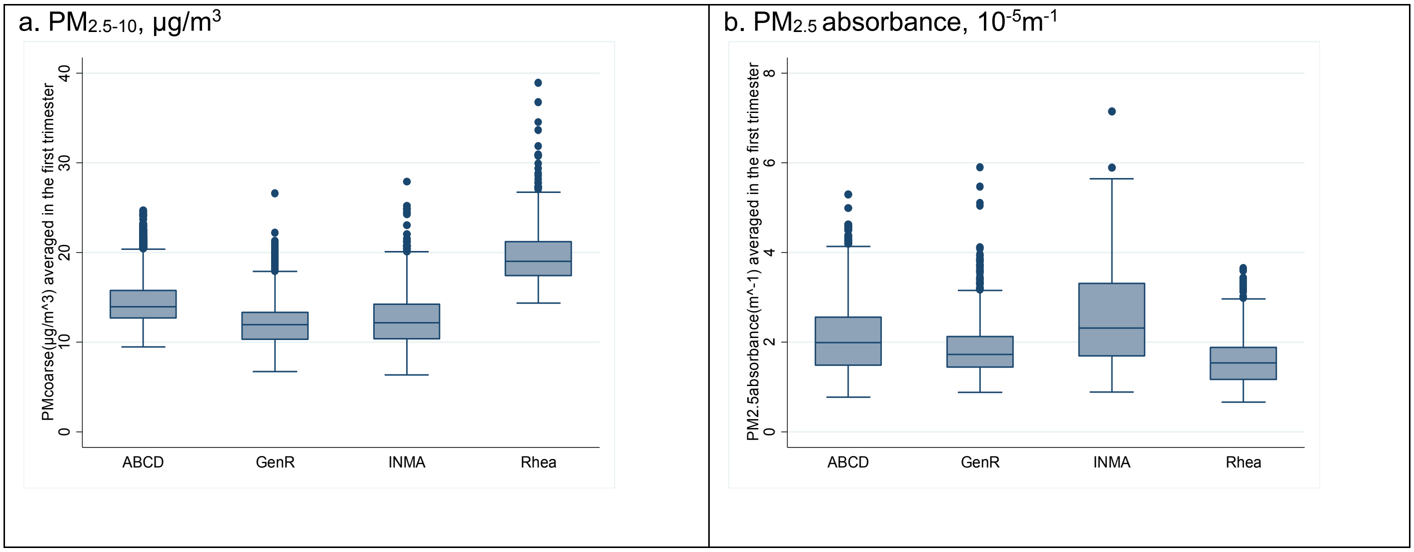

ABCD: Amsterdam Born Children and their development; GenR: the Generation R Study; INMA: Infancia y Medio Ambiente; $PM_{2.5-10}$: Particulate matter between 2.5 and 10µm; $PM_{2.5}$ absorbance: Reflectance of $PM_{2.5}$ filters

Data are presented as available in each cohort. Data on PM were only available in Sabadell sub-cohort of INMA.

© 2019 Ghassabian A et al. *JAMA Network Open.*

eFigure 2. Exposure to $PM^{2.5-10}$ and $PM_{2.5}$ Absorbance in the First Trimester and Thyroid Function During Pregnancy

a. $PM_{2.5-10}$ (per Δ5 µg/m^3) exposure and hypothyroxinemia

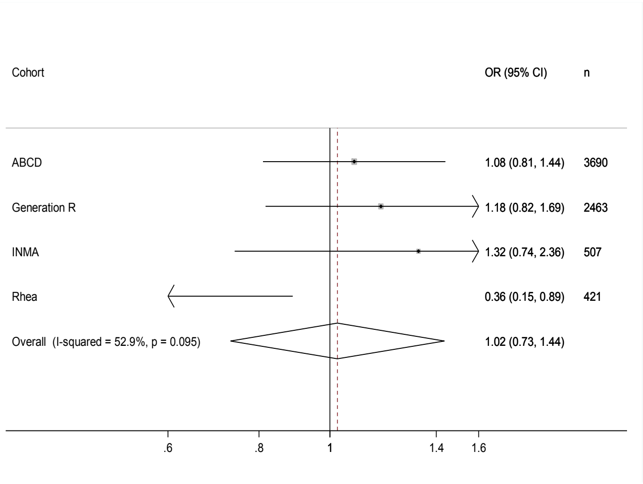

b. $PM_{2.5}$ absorbance (per Δ $10^{-5}m^{-1}$) exposure and hypothyroxinemia

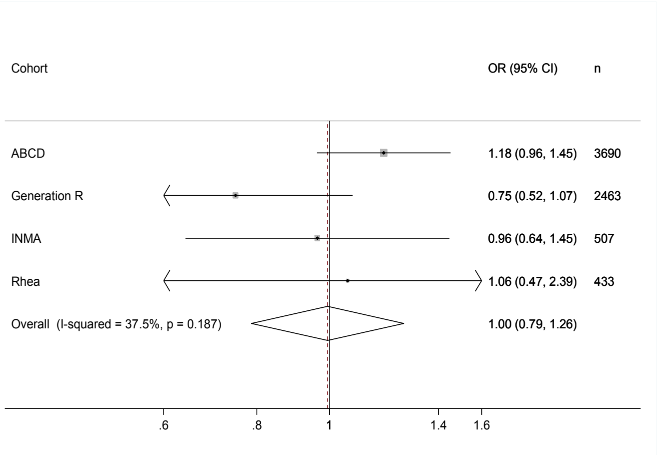

c. $PM_{2.5-10}$ (per Δ5 µg/m^3) exposure and high TSH

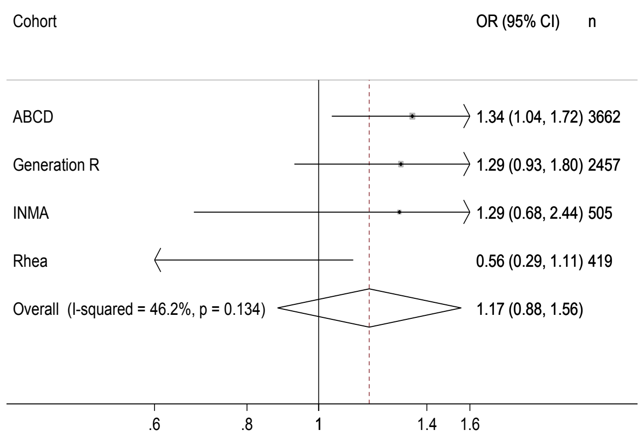

d. $PM_{2.5}$ absorbance (per Δ $10^{-5}m^{-1}$) exposure and high TSH

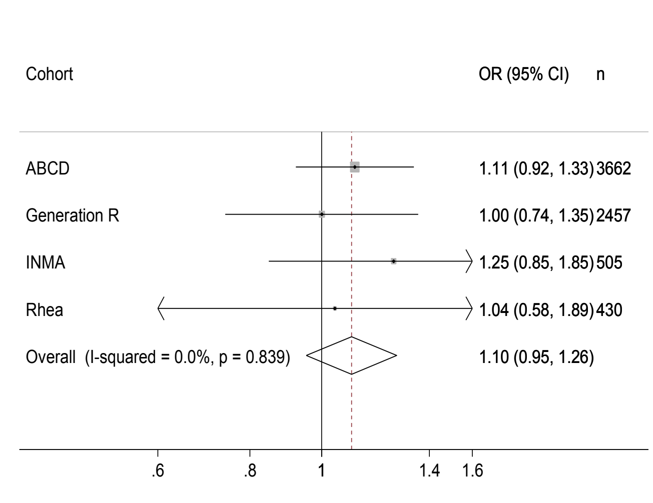

ABCD: Amsterdam Born Children and their development; CI: Confidence interval; OR: Odds ratio; P: P value of heterogeneity; $PM_{2.5-10}$: Particulate matter between 2.5 and 10µm; $PM_{2.5}$ absorbance: Reflectance of $PM_{2.5}$ filters; T4: Thyroxine; TSH: Thyroid stimulating hormone.

Odds ratios (95%CI) are shown for the associations between $PM_{2.5-10}$ exposure and hypothyroxinemia (a), $PM_{2.5}$ absorbance exposure and hypothyroxinemia (b), $PM_{2.5-10}$ exposure and high TSH (c), and $PM_{2.5}$ absorbance exospore and high TSH (d), estimated using random-effects meta-analysis by cohort (ABCD, Generation R, INMA, Rhea, and Project Viva).

We defined hypothyroxinemia as free T_4 below the 5th percentile of cohort distribution despite normal TSH. High TSH was defined as values > 95th percentile. I^2 refers to the percentage of the total variability due to between-areas heterogeneity. P value of heterogeneity was estimated using the Cochran's Q test.

Models were adjusted for pregnant women's age at enrollment, educational level, country of birth, gestational age at thyroid measurement, history of smoking, alcohol intake during pregnancy, socioeconomic status, marital status, parity, and pre-pregnancy body mass index. Data on PM were only available in Sabadell region of INMA. Data are presented as available in each cohort.

© 2019 Ghassabian A et al. *JAMA Network Open.*
